# Preface to the Second Edition

**Published:** 1896

**Authors:** Constantine Hering


### PREFACE TO THE SECOND EDITION.

Dr. Hering's monograph upon Typhoid Fever was published many years ago as a part of his famous system of "Analytical Therapeutics." This system was never finished, and the fragment containing Typhoid Fever was about all that remained in practical use, of the portion published.

For some years it has been out of print.

The demand for it, however, has by no means ceased. Those who have been unsuccessful in obtaining it, those who by continual use have worn out their own copies have repeatedly desired that it should be republished by *The Homœopathic Physician.*

The remarkable success of homœopathic practice in bringing about a happy recovery from Typhoid Fever, where pains are taken to give the similar remedy, has impressed the editor with the need the physicians of our school have for every possible assistance in selecting the true simillimum, the better to insure this success.

This thought has decided the editor to gratify these wishes, and give to the profession the much-desired book.

Through the kindness of Dr. A. W. Holcombe, of Kokomo, Indiana, who was willing to sacrifice his copy for the good of the cause, we are enabled to present this publication to the profession.

The original was a large quarto with double columns which made the book much too cumbersome for general use. In reprinting, it has been deemed advisable to reduce it to the present size of page in order that it shall be more serviceable for reference at the bedside of the patient.

In other respects no important changes, additions, or subtractions have been made to the text, so that the reader may rely upon the accuracy of this reproduction of Hering's valuable work.

WALTER M. JAMES, M. D.,
Editor of *The Homœopathic Physician.*

P. S.—The marginal references as described in the preface at page 8 had to be altered, since they were arranged for a quarto page with double columns. The figures as now inserted will correspond with page and paragraph.

